# Clinical implement of mp-tNGS and hc-tNGS in pathogenic diagnosis of lower respiratory tract infections

**DOI:** 10.1016/j.ebiom.2024.105346

**Published:** 2024-09-24

**Authors:** Ying Fu, Hua Zhou

**Affiliations:** a Department of Clinical Laboratory, Sir Run Run Shaw Hospital, Zhejiang University School of Medicine, Hangzhou, Zhejiang Province, 310016, China; b Department of Respiratory and Critical Care Medicine, The First Affiliated Hospital, Zhejiang University School of Medicine, Hangzhou, Zhejiang Province, 310003, China

Lower respiratory tract infections (LRTIs) represent a significant global health challenge, contributing substantially to both morbidity and mortality.^1^ Accurate and timely diagnosis of pathogens and drug resistance is essential for effective clinical diagnosis and appropriate treatment, especially in severe infections or infectious diseases that pose a risk of widespread transmission. The range of pathogens involved in LRTIs is diverse, including Gram-positive and negative bacteria, atypical bacteria, viruses, fungi, and parasites. This complexity poses significant challenges for diagnosis, making advanced techniques crucial for effective management.

Shotgun metagenomic next-generation sequencing (mNGS) has emerged as a powerful tool in pathogen detection due to its ability to analyze a broad spectrum of microorganisms in a sample. However, it is expensive and the sensitivity in pathogen detection is heavily influenced by the abundance of nucleic acid originating from the host. This has led to the development of enrichment strategies towards the pathogens, such as targeted NGS (tNGS), which aims to improve detection efficiency and reduce costs. Among the tNGS techniques, multiplex PCR-based tNGS (mp-tNGS) and hybrid-capture-based tNGS (hc-tNGS) have shown promises. The mp-tNGS works by using specific primers to amplify pathogen-specific sequences in a sample, allowing for the detection of multiple pathogens. The key challenge with mp-tNGS is the design and optimization of the multiplex reaction, especially with a broad range of primers that target specific LRTI pathogens. Li and colleagues have demonstrated that their mp-tNGS pipeline enriched 153 pathogens, covering more than 95% of respiratory infections.^2^ On the other hand, hc-tNGS enriches pathogens by using sequence-specific DNA or RNA probes that can hybridize with pathogen-specific nucleic acid. This technique allows for the detection of a large panel of pathogens simultaneously.^3^ However, studies comparing the analytical and diagnostic performances of mp-tNGS, hc-tNGS, mNGS, and conventional microbiological tests (CMTs) are lacking. Such comparisons would be critical for making informed clinical decisions on which tests to use.

A recent study published in *eBioMedicine* by Yin and colleagues has advanced the field by developing and evaluating mp-tNGS and hc-tNGS methods. Their study demonstrated that both methods were efficient in detecting pathogens across a broad spectrum (198 and 3060 pathogens, respectively), with shorter turnaround times (10.3 and 16 h), reduced costs (one-quarter and one-half of mNGS costs, respectively), and increased detection limits (50–450 CFU/mL (copies/mL) for all target microorganism).^4^ The clinical validation involved 229 retrospective and 251 prospective bronchoalveolar lavage fluid (BALF) samples, and the results showed that mp-tNGS and hc-tNGS achieved 100% concordance in intra- and inter-assay consistencies. These methods demonstrated high accuracy with sensitivities of 86.5% and 87.3% (compared to 85.5% for mNGS) and specificities of 90.0% and 88.0% (compared to 92.1% for mNGS). The detection rates for common pathogens were 84.3% and 89.5% (compared to 88.5% for mNGS), which were significantly higher than conventional microbiological tests. Notably, their prospective research also classified pathogen results based on both laboratory and clinical data, enhancing the interpretation of tNGS reports and providing valuable guidance for clinical decision-making.

In July 2023, the World Health Organization (WHO) endorsed tNGS as a new diagnostic technology for detecting drug-resistant tuberculosis (TB). This endorsement underscores the method's value in identifying TB and detecting drug-resistant point mutations.^5^^,^^6^ Despite this recognition, challenges remain. The complexity of pathogens in respiratory specimens necessitates laboratory reports that differentiate between pathogenic bacteria, opportunistic pathogens, and colonizing microorganisms. Furthermore, when establishing tNGS methodologies, a larger panel is not always better. It is essential to compare existing diagnostic techniques for LRTIs and clinical evidence to refine the sensitivity and specificity of the methodology. Yin and colleagues have provided a comprehensive comparative analysis in this field. If multi-center validation and wider dissemination are achieved, the method's potential could be significantly expanded. Another critical aspect of tNGS is its ability to detect antimicrobial resistance gene. Compared to mNGS, tNGS offers enhanced detection of resistance genes. Although Yin's study includes primers for detecting resistance genes with mp-tNGS, it does not compare the detection capabilities in this context. Point mutations are particularly important for antimicrobial suseptability tests, such as rifampicin-resistant TB. Theoretically, hc-tNGS has the potential to excel in this area, playing a crucial role in future developments. However, the mp-tNGS method discussed here has yet to address this key aspect in its design.

In summary, Yin's study confirmed the high sensitivity of mp-tNGS and hc-tNGS for pathogen detection in BALF specimens from patients of LRTIs. Considering its lower cost and shorter TAT time, these two methods could be considered as important tools for detecting pathogens of LRTIs. While, the potential diagnostic efficiency of other specimens (such as cerebrospinal fluid and plasma) and drug resistance genes have yet to be validated by clinical research.

## Contributors

Literature search: Y. F and H. Z; Data interpretation: Y. F. and H. Z; Writing: Y. F and H. Z. Both authors read and approve the final manuscript.

## Declaration of interests

The authors declare no conflict of interest.
